# Contribution of uric acid to cancer risk, recurrence, and mortality

**DOI:** 10.1186/2001-1326-1-16

**Published:** 2012-08-15

**Authors:** Mehdi A Fini, Anthony Elias, Richard J Johnson, Richard M Wright

**Affiliations:** 1 Department of Medicine Pulmonary Division and Cardiovascular Pulmonary Research Laboratory, University of Colorado Denver, Anschutz Medical Campus, V20, Room 3104, Mail stop C-322 12850 East Montview Boulevard, Aurora, CO, 80045-0511, USA; 2 Martha Cannon Dear Professor of Medicine, Medical Director, Breast & Sarcoma Programs, Associate Director of Cancer Center for Clinical Sciences, AOP-3115, MS F-724 1635 Aurora Court, Aurora, CO, 80045, USA; 3 Tomas Berl Professor of Medicine, Chief, Division of Renal Diseases and Hypertension, University of Colorado Denver, Anschutz Medical Campus, Mail Stop C281, 12700 E 19th Ave, Room 7015, Aurora, CO, 80045-0511, USA; 4 Department of Medicine, Pulmonary Division and Webb-Waring Center, University of Colorado Denver, Anschutz Medical Campus, V20, Room 3105, Mail stop C-322 12850 East Montview Boulevard, Aurora, CO, 80045-0511, USA

**Keywords:** Cancer, Obesity, T2DM, Metabolic syndrome, Uric acid

## Abstract

Two risk factors for the development and progression of cancers that are amenable to life style modification are chronic inflammation and the metabolic syndrome. This review proposes two new targets that may mechanistically integrate inflammation and metabolic syndrome, have been largely ignored, and are known to be druggable. Recent evidence has demonstrated that elevated serum uric acid (hyperuricemia) is associated with excess cancer risk, recurrence, and mortality. Although uric acid (UA) can function as a systemic antioxidant, its pro-inflammatory properties have been postulated to play an important role in the pathogenesis of cancer. Furthermore, obesity, Type 2 Diabetes Mellitus (T2DM), and the metabolic syndrome (MetS) are also associated with excess cancer, chronic inflammation, and with hyperuricemia, suggesting that UA may represent an important link between these disorders and the development of cancer. While pharmacological modulation of hyperuricemia could in principal augment anti-cancer therapeutic strategies, some cancer cells express low intracellular levels of the enzyme Xanthine Oxidoreductase (XOR) that are associated with increased cancer aggressiveness and poor clinical outcome. Thus, systemic pharmacological inhibition of XOR may worsen clinical outcome, and specific strategies that target serum uric acid (SUA) without inhibiting tumor cell XOR may create new therapeutic opportunities for cancer associated with hyperuricemia. This review will summarize the evidence that elevated SUA may be a true risk factor for cancer incidence and mortality, and mechanisms by which UA may contribute to cancer pathogenesis will be discussed in the hope that these will identify new opportunities for cancer management.

## Review

### Hyperuricemia and cancer

UA is derived exclusively from the oxidation of xanthine and hypoxanthine by XOR [1], and pharmacological inhibition of XOR has been used extensively for the management of hyperuricemic disorders such as gout, nephrolithiasis, some cases of acute and chronic kidney disease, ischemia-reperfusion disorders, and others [2,3]. In 1982 UA was hypothesized by Ames *et al.* to provide a primary defense against human cancer based upon its capacity to scavenge singlet oxygen, its capacity to inhibit lipid peroxidation, and its high serum concentration in humans [4]. Extensive support for the physiological antioxidant function of UA was generated in the ensuing dozen years since publication of this hypothesis, and the protective antioxidant properties of UA have been identified in many different organ systems [5]. Nonetheless, elevated serum urate, the dominant monosodium form of UA at physiological pH, was found to exhibit strong statistical association with increased premature cancer death in both men and women [6-8] suggesting a more complex role for UA in cancer biology than that of a general antioxidant (Table 1).

**Table 1:** References demonstrating the specific association of SUA with cancer risk, recurrence, and mortality

**Reference**	**Risk category**	**Gender**	**Cancer type**	**SUA measurement**
Petersson et al., 1983	Unselected	M	All	Concurrent
Petersson et al., 1984	Unselected	M	All	Concurrent
Levine et al., 1989	Unselected	F	All	Prospective
Kolonel et al., 1994	Unselected	M	Prostate	Prospective
Korenga et al., 2005	ABCG2	M/F	Renal	Prospective
Tsimberidou et al., 2005	MetS/Obesity	M/F	All	Prospective
Shin et al., 2006	Unselected	M/F	All	Concurrent
Giovannucci, 2007	MetS	M/F	Colon	Prospective
Hu et al., 2007	ABCG2	M/F	B-Cell Lymphoma	Prospective
Rose et al., 2007	MetS	F	Breast	Concurrent
Strasak et al., 2007a	Unselected	F	All	Prospective
Strasak et al., 2007b	Unselected	M	All	Prospective
Boffetta et al., 2009	Gout	M/F	All	Prospective
Becker et al., 2009	Obesity	M/F	All	Concurrent
Strasak et al., 2009	Unselected	M/F	All	Prospective
Bjorge et al., 2011	MetS	F	Breast	Concurrent
Hammarsten et al., 2011	MetS	M	Prostate	Prospective
Panero, et al., 2011	T2DM	M/F	All	Prospective
Siddiqui, 2011	MetS	M/F	Colorectal	Prospective
Wang, et al., 2011	ABCG2	M/F	Leukemia	Prospective

In humans, normal SUA levels are commonly between 178 and 360 μM, (3 and 6.8 mg/dl) with higher levels found in males and postmenopausal females than in premenopausal females. Frank hyperuricemia (SUA levels > 360 μM) can reach levels of 700 μM or higher and are associated with increasing risk for gout and acute kidney injury arising from the deposition of monosodium urate (MSU) crystals in the renal tubules and interstitium [9]. Mechanisms inducing MSU crystal formation and deposition are complex and not simply an automatic consequence of hyperuricemia [10]. More recently there has been increasing interest that high to high normal levels of SUA (310–330 μM), below those associated with MSU crystal deposition, may have contributory roles in acute renal injury [11], chronic kidney disease [12,13], hypertension [14], cardiovascular disease (CVD) [15-17], and MetS [18-20]. While hyperuricemia is increased by age, menopause, alcohol consumption, and other dietary factors, as a component of MetS, hyperuricemia is associated with increased risk of colorectal, breast, prostate, and other cancers [21-25].

Although early reports established an epidemiological association between SUA and age, sex, and increased death due to cancer of all types [6,7], numerous confounding factors including diet, alcohol consumption, and underlying co-morbidity disorders like diabetes, CVD, or MetS may have been responsible for the observed association. Furthermore, cancer itself could promote hyperuricemia through cancer related cell death rather than being an independent risk factor for the development of cancer. It is important to recognize in passing that both of these early reports and subsequent studies that observed an association between SUA and death due to cancer also observed an inverse relation with serum cholesterol [6,7,26]. While these publications did not explain the inverse association with cholesterol, it is possible that the cachexia of cancer, which is associated with reduced fat stores and muscle wasting, releases glutamate and glutamine that can increase SUA levels, and indeed elevated SUA has been associated with sarcopenia [27]. On the other hand, *in vitro* analysis of liver tissue slices demonstrated that UA itself inhibited cholesterol biosynthesis upstream of mevalonic acid, possibly by inhibition of hydroxymethylglutaryl CoA reductase [28], suggesting a potentially broad effect of UA on cholesterol level.

Data from prospective studies, however, do suggest that SUA may predict the development of cancer. After adjusting for a large number of confounding factors, Levine *et al.*[8] observed that SUA measured prospectively at baseline before the development of cancer was significantly associated with all site cancer mortality over 11.5 years of follow-up in women aged 55–64, and was, therefore, unlikely to reflect hyperuricemia developed secondarily to the development of cancer. A similar prospective analysis in Japanese men used the Cox Proportional Hazard Ratio (HR) to identify an association of elevated SUA with the risk for development of prostate cancer over a period of ten years following baseline measurement [29]. Similarly, the increase in incident prostate cancer observed in a Swedish cohort of MetS males was associated with high SUA and insulin levels, and both SUA and insulin were significant parallel prospective markers of risk for prostate cancer. Indeed, a SUA level above 358 μM was found by binary regression analysis to be an independent and significant (p < 0.04) prospective risk factor for incident prostate cancer [21]. Further and much larger prospective studies conducted on both male and female European cohorts confirmed that high SUA (>6.71 mg/dl in men and >5.41 mg/dl in women) measured at baseline was an independent risk factor for death from all cancers compared to high normal SUA (4.6 mg/dl) [30,31]. These studies achieved very high significance (adjusted HR, p < 0.0001) comparing death ten years after measurement of antecedent SUA, and as also noted by Levine [8], the strongest association between baseline SUA and cancer death was achieved in the older patient quartile. These studies were important as well because SUA obtained at baseline was derived from apparently healthy people.

Important advances in statistical methods were subsequently employed to identify both the dose dependence and time varying association of SUA and risk for all site cancer mortality [32]. Finely stratified SUA obtained at baseline and with 18.5 years of follow-up demonstrated the impact of SUA on risk for cancer mortality at both high and very low UA levels (J-Shaped dose–response curve). This study was the first to identify SUA as both a time dependent co-variant risk factor for overall cancer incidence and one exhibiting a clear dose–response to baseline SUA. Furthermore, amongst patients already exhibiting terminal end stage cancer, weekly measurement of SUA from the day of admission to cancer associated death revealed that high SUA (>7.2 mg/dl) significantly and independently predicted reduced survival time [26], revealing yet another dimension to the risk for cancer mortality by elevated SUA.

Collectively, these data identify elevated SUA to be independently and significantly associated with the risk for all site cancer incidence and mortality when measured in advance of the development of cancer. While the incidence of cancer and cancer mortality observed in hyperuricemia do not support the hypothesis that the antioxidant properties of UA provide anti-cancer defense in humans [4], the increment in cancer seen by Strasak *et al.*[32] at low dose SUA may suggest a protective effect of SUA that is optimal near the normal human levels of SUA. Furthermore, since low SUA may also reflect poor nutrition, these observations raise the possibility that a low SUA might have been a sentinel sign prior to the recognition of cancer.

### SUA, inflammation, and cancer

The association of elevated SUA with increased cancer risk and mortality predicts that diseases associated with hyperuricemia would also exhibit excess cancer risk and mortality. Obesity, T2DM, insulin resistance, hypertension, MetS, and gout comprise a cluster of syndromes that are associated with hyperuricemia, chronic inflammation, and activated innate immunity [33,34] that may be mediated in part by UA [35-38]. Elevated SUA has been associated with prepubertal obese children presenting with insulin resistance [39], and it was identified as a strong and reliable biomarker of MetS in obese young women [40]. Meta-analyses have consistently shown that SUA is a potent independent predictor of hypertension, insulin resistance, and diabetes [41,42]. Furthermore, confirmatory factor analysis of selected variables has identified UA as a single common factor linking four of the core components in the definition of MetS including the HOMAR-IR measure of insulin resistance, mean arterial pressure, the ratio of serum triglycerides to HDL-cholesterol, and waist circumference [43]. Experimental evidence is building that this factor could indeed represent UA, and recent analysis has identified its potential phyiological role in T2DM and related disorders [44].

Excess cancer risk and incidence have been associated with obesity, T2DM, insulin resistance, MetS, and gout in large epidemiological analyses. For example, increased all site cancer incidence has been observed in a large cohort of both male and female gout patients, and increased overall cancer risk persisted in this population throughout the 5 to 15 year follow-up from the time of gout diagnosis [45]. The British Heart Disease and Diabetes Indicators Screened Cohort study (HDDRISC) identified a cluster of biomarkers comprising numerous inflammatory markers including SUA. SUA independently and as a component of the biomarker cluster was predictive of all site cancer mortality in T2DM patients through 21.5 years of follow-up [46]. This study also confirmed the significant inverse relationship between cancer mortality, SUA, and serum cholesterol in diabetic patients. In a smaller but significant study of T2DM patients from Northern Italy, high SUA was found to be strongly associated with increased mortality that was almost entirely the result of all site neoplastic disease. Statistically significant increased cancer risk and mortality was observed for SUA levels above 226 μM that persisted through the highest SUA quartile [47]. These data confirmed a massive study conducted earlier by the Cancer Prevention Study II of 1.2 million US men and women that identified diabetes as an independent predictor of mortality from cancer of the colon, pancreas, breast, liver, and bladder [48]. Although the CPS-II did not compile data on SUA *per se*, a wide range of dietary and lifestyle covariant parameters were included in the analysis that have been found independenly to promote hyperuricemia, including obesity, lack of exercise, western diet, red meat consumption, and alochol consumption. Obesity, T2DM, insulin resistance, and MetS have been specifically associated with increased risk of breast cancer (BC) [22,36,49-52], BC recurrence [53,54], and more aggressive tumor biology [55,56]. The Triple Negative Breast Cancer subtype that exhibits poor prognosis, excess recurrence and metastasis, and worse chemotherapeutic response was significantly more prevalent in patients exhibiting MetS [36,57,58].

Chronic low grade inflammation is an underlying component of obesity, T2DM, insulin resistance, and MetS that is mediated in part by the pro-inflammatory properties of UA [35-38]. UA has been found to promote inflammation in two ways: as an MSU crystal or as a soluble factor. Recent data have consolidated the idea that the MSU crystal functions as a “danger signal” recognized by Toll receptor-4 [9,10,59] and contributing to many inflammatory disorders. Dead and dying cells have been postulated as one source of UA in an inflammatory microenvironment, and activation of Toll receptors by MSU crystals has been found to stimulate leukocyte pro-inflammatory cytokine production [59,60]. On the other hand, in its soluble form, UA was found to enter cells where it activated MAP kinases (p38 and ERK), stimulated NFĸB, and induced expression of inflammatory mediators including MCP-1 and C-Reactive Protein (CRP) [61-64]. These effects were likely mediated by NADPH oxidase induced oxidative stress inside the cell [61,62,65,66]. Importantly, additional *in vivo* studies further demonstrated that lowering UA pharmacologically improved inflammation both in laboratory animals and in patients with chronic kidney disease [13,67]. Inflammatory processes induced by UA in its soluble form require the function of transport proteins (GLUT9, URAT1, and others) that translocate soluble UA into cells activating wide genetic reprogramming and is therefore mechanistically distinct from Toll receptor activation by MSU crystals [68,69]. Adiponectin, C-Reactive Protein, and Leptin are key components of the chronic inflammatory environment that have been associated with elevated SUA levels and cancer.

### Adiponectin, SUA, and cancer

Adiponectin is an anti-inflammatory protein whose levels are reduced in obesity, T2DM, insulin resistance, and MetS [70] and when reduced it has been associated with increased risk of diverse cancers [71-73]. Reduced circulating adiponectin was associated with the risk for hypertension and renal injury, and it was inversely associated with Leptin and CRP levels [74]. Low adiponectin levels were found to be inversely associated with high SUA in both young obese children and in adult women presenting with MetS [70,75]. Therefore, it is highly significant that in mouse models of MetS UA *per se* was found to specifically reduce serum adiponectin level [67], suggesting a functional link between SUA and adiponectin expression.

High circulating levels of adiponectin have been strongly linked to improved BC risk and outcome [23,36,76], and adiponectin treatment was found to attenuate both cancer cell proliferation and mammary tumor progression in xenograft analysis of aggressive BC cells in mice [77]. The antitumor properties of adiponectin may be in part related to its capacity to inhibit tumor angiogenesis [78]. On the other hand, low circulating adiponectin level has been a commonly observed risk factor for BC [53,79-81]. Although most commonly associated with post-meopausal BC [23,76,80], low serum adiponectin was associated with increased BC risk in both pre- and post-menopausal Japanese patients [81]. Low circulating adiponectin was associated with increased BC risk overall [53,79-81], BC recurrence and metastasis [53,82], and increased BC mortality [79]. In transgenic mouse models of mammary tumorigenesis that utilized the MMTV driven polyoma middle-T antigen, haploinsufficiency of adiponectin was found to accelerate mammary tumor onset and increase aggressiveness and tumor development [83]. Furthermore, increased lung metastases were observed by a different group of investigators using the same model of mammary tumorigenesis [84]. This group made the apparently contradictory observation that haploinsufficiency of adiponectin produced a delayed angiogenic response, an observation that was explained as a potential angio-mimetic property of adiponectin acting in the early, but not late, stages of tumor vascularization [85]. Current data indicate that adiponetin may in part exert its effects on mammary tumor cells by inhibition of Wnt signalling, Akt activity, and the tumor suppressor LKB1 [77,82]. Furthermore, loss of adiponectin has been found to promote hyperactivation of PI3K/Akt phosphorylation and signalling that was associated with increased proliferation of mammary tumor cells [83]. Together, these data are consistent with a role for high SUA in depressing circulating adiponectin level that is in turn associated with increased tumorigenesis, tumor size, and metastasis.

### C-reactive protein, SUA, and cancer

Elevated SUA has been specifically associated with high circulating levels of the pro-inflammatory mediator C-Reactive Protein (CRP) as well as the pro-inflammatory mediators IL-6 and TNFα in a large cross-sectional European cohort [86]. Co-expression of high CRP and elevated SUA was identified in patients with T2DM [87] or MetS [88-90] where it was specifically associated with CVD or insulin resistance. Elevated levels of CRP were positively associated with increased BC risk and risk of cancer death [36,91], and they were positively correlated with SUA levels in early and advanced stages of BC [92]. High circulating levels of CRP were also associated with increased risk for colorectal [93], lung [94], gastric [95], and renal cell cancers [96]. Significantly, pharmacolological inhibition of XOR with allopurinol concomittantly reduced both SUA and CRP in patients with either normal renal function or in patients expressing chronic kidney disease [13,97,98], and direct support for a functional role of UA in the production of CRP was obtained in vascular smooth muscle cells and endothelial cells where UA stimulated secretion of CRP as well as MCP-1 [61,62].

Despite these strong associations between CRP, SUA, and the risk for diverse types of cancer, CRP is unlikely to be a specifc cause of cancer. Analysis of the four most common single nucleotide polymorphisms (SNPs) found in the CRP gene that were associated with increased circulating CRP (95% CI of 58-87% increase) revealed that there was no increase in cancer risk or incidence associated with these SNPs [99]. This study employed a large European cohort of 10,215 subjects and demonstrated an insignificant odds ratio for cancer associated with doubling of CRP level of 0.94 (95% CI of 0.81 to 1.08). While these data indicated that elevated CRP *per se* was unlikely to be a cause of cancer, they did identify CRP as an important prognostic biomarker of outcome that revealed an underlying inflammatory process associated with high SUA levels [100].

### Leptin, SUA, and cancer

Leptin has been described as the missing link between hyperuricemia and obesity, MetS, T2DM, and related disorders [101,102]. Leptin is the product of the Obese gene (ob), and loss of function mutations in mouse and human ob genes results in profound obesity and T2DM [103,104]. Deficiency in receptors for leptin (LepR^db-db^ and Lepr^db-lb^) in mice likewise result in obesity, diabetes, pre-diabetes, and MetS [67,105]. Thus, it has been postulated that while leptin and leptin receptor signaling act to suppress food intake and maintain long-term energy balance, the excess serum leptin found in obesity may reflect a state of leptin resistance [104].

Leptin, CRP, and SUA are directly correlated in patients presenting with MetS [40,74,106-108], and inversely correlated with adiponectin level [75,107]. Leptin has been postulated to be a key mediator linking obesity and hyperuricemia as a potential regulator of SUA level. Statistical models have revealed strong positive association between circulating leptin and SUA, and in multifactorial regression analyses serum leptin could explain 34-42% of the variance in SUA in both men and women in a hypertensive cohort of Turkish patients [101]. Leptin and SUA were strongly associated in healthy and T2DM subjects [102], in patients presenting with MetsS, central obesity, and insulin resistance [109,110], and indeed in a Japanese cohort of patients with gout, gout was associated with excess MetS compared to normal controls and was strongly associated with elevated leptin and SUA [111].

There is some evidence that the rise in uric acid may be the consequence of elevated leptin levels because loss of leptin receptor signaling also engenders hyperuricemia [67]. Fruehwald-Schultes *et al.*[102] have suggested a mechanism that may in part explain this observation. They have postulated that leptin may directly impair UA excretion in the kidney and that in obesity elevated leptin levels may impair renal clearance of UA resulting in hyperuricemia. Hyperuricemic, leptin receptor deficient mice also show excessive hepatic XOR expression suggesting that leptin receptor signaling may also down regulate hepatic XOR expression [67]. Thus, leptin may affect SUA at both the level of production and renal clearance. Taken together, these results suggest an additional link between SUA and cancer: with leptin regulating SUA levels in subjects with obesity, MetS, and T2DM.

There is also experimental evidence that UA may have a role in mediating leptin resistance. Recent studies have reported that elevated SUA precedes the development of MetS in humans [42], suggesting that it might precede the development of leptin resistance. Experimental studies found that lowering SUA pharmacologically improved features of MetS in rats in response to fructose feeding, including the development of insulin resistance, hypertriglyceridemia, and elevated blood pressure [19]. More recently, a pilot study found that lowering SUA in fructose-fed rats also reduced leptin expression in visceral fat tissues [112]. Further studies will be required to determine if the reduced leptin expression would also translate into reduced leptin resistance.

The pro-tumorigenic role of leptin on breast, colon, prostate, and ovarian cancer in patients with obesity, MetS, and T2DM was recently reviewed [85,113,114]. Briefly, mounting evidence suggests that the leptin to adiponectin ratio is sensitive to states of obesity, T2DM, and MetS and that the ratio is a key clinical determinant of cancer tumorigenesis and/or carcinogenesis with elevated leptin being associated with greater cancer risk and poor outcome. The pro-tumorigenic effect of leptin appears to be mediated in part by its stimulation of cancer cell proliferation via ERK, JNK, and STAT3 pathways. While leptin is an apparent growth factor for BC that is elevated in BC patients [36,115], its mechanism of tumor promotion remains equivocal [36]. Importantly, leptin receptors are expressed on many cancer cells including those of the breast [116], and exposure of MCF-7 BC cells to leptin induced cell proliferation that was mediated in part by activation of MAPK [117]. As described below, we speculate that leptin may further stimulate proliferation and tumorigenesis by down-regulating cancer cell XOR.

### UA transport and cancer

Homeostasis of SUA in humans is maintained by the bidirectional flux of SUA in renal proximal tubule epithelial cells. Presently, no less than 10 plasma membrane transport proteins have been found to contribute to UA movement into and out of the renal proximal tubule epithelial cells [68]. Roles for the Organic Anion Transport (OAT) proteins OAT-1, -3, -4, 10, ABCG2, NPT1/4, MRP4, URAT1, and GLUT9 in renal UA homeostasis and extra-renal distribution are only partially understood. For many of these, a large array of accessory proteins has been identified that are required for co-transport function [68]. The two major renal transporter proteins identified to date are the high affinity transporter URAT1 (SLC22A12) that mediates uptake of urate from the urine into the renal proximal tubular cell, and GLUT9 (SLC2A9/URATv1) which is a voltage-sensitive uniporter that mediates the export of UA from the proximal tubular cell into the circulation [118,119]. The association of URAT1 or GLUT9 with cancer remains largely untested at the present time.

The ABCG2 locus encoding a member of the ATP-binding cassette transporter proteins, also known as the Breast Cancer Resistance Protein (BCRP), has been intensively studied for its role in resistance to cancer chemotherapy. ABCG2 is an important efflux transporter of xenobiotic compounds, including many antineoplastic drugs, and its inhibition or mutation can improve cancer chemotherapy [120]. Genome wide association studies (GWAS) for hyperuricemia identified ABCG2 as tightly associated with hyperuricemia and gout [121-124]. Functional analysis confirmed the principal role of ABCG2 in normal physiology, independent of drug efflux, as that of UA efflux. These studies identified several SNP associated with the ABCG2 locus that were functionally involved in UA efflux. In particular, SNP rs2231142 generated the C421A DNA mutation causing the Q141K amino acid substitution. Q141K both reduced ABCG2 expression and blocked UA efflux, and it is a common polymorphism causing gout and hyperuricemia in Caucasian, Black, Japanese, and Chinese populations [121-124].

Prospective analysis of the C421A polymorphism in untreated patient populations, those not undergoing any chemotherapeutic drug treatment, demonstrated significant and markedly increased risk for developing nonpapillary renal cell carcinoma [125], an increased risk and poor survival prognosis for patients with diffuse large B-cell lymphoma [126], and increased risk and poor survival prognosis for patients with acute leukemia [127]. These data strongly support the premise that hyperuricemia can be an important risk factor for cancer incidence and mortality, and they underscore the importance of conducting more expansive analysis of other UA transport proteins in diverse cancer settings.

**Figure 1:**
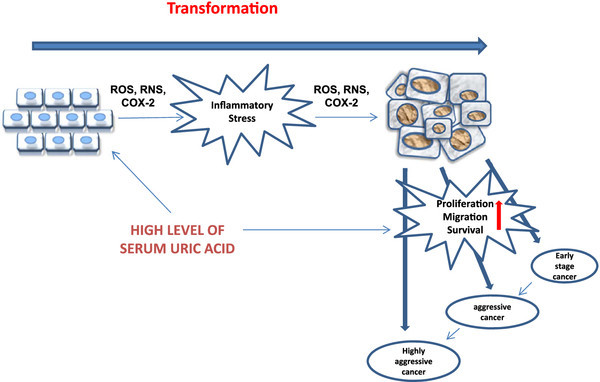
**Hyperuricemia contributes to tumorigenesis by promoting both transformation and tumor cell proliferation, migration, and survival.** High levels of extracellular UA present in the serum or in the local microenvironment of tumor cells exerts many pro-inflammatory effects that contribute to tumorigenesis. While extracellular UA has antioxidant effects that may protect normal cells from transformation, entry of UA into cells can generate inflammatory stress that arises from the effects of intracellular UA on ROS/RNS generation and COX-2 activation. Stimulation of cancer cells by UA further promotes proliferation, migration, and survival that mediates progression from early stage cancer to highly aggressive cancer.

Together these data suggest a model by which both extracellular UA and intracellular UA may collaborate in transformation toward a highly aggressive cancer (Figure 1). Inflammatory stress induced by elevated intracellular UA may promote transformation, while elevated extracellular UA may further stimulate tumor cell proliferation, migration, and survival contributing to the development of highly aggressive cancer.

### Tumor cell XOR, UA, and cancer

Although the increased cancer burden associated with hyperuricemia in obesity, T2DM, and MetS has suggested the relevance of modulating XOR activity in cancer therapy, presently available strategies may be inappropriate for many types of cancer. Allopurinol and febuxostat are FDA approved systemic pharmacological inhibitors that block XOR activity in all cells of the body [2,128]. However, inhibition of XOR in tumor cells *per se* is a potentially confounding factor that limits current strategies for the pharmacological control of SUA in cancer management.

Decreased or absent tumor cell XOR has been observed in the most aggressive human breast cancer [129], gastric cancer [130], colorectal cancer [131], ovarian cancer [132], non-small-cell lung cancer [133], and in rat hepatic cancer [134,135]. For breast, gastric, colorectal, ovarian, and lung cancers in humans the decreased XOR activity was associated with worse clinical prognosis and unfavorable outcome (reduced survival). Poor XOR expression was also associated with poorly differentiated breast, gastric, and colorectal cancers and was associated with over two fold increased risk of distant metastasis. While it has been suggested that the decreased purine catabolism and increased activity of salvage pathway enzymes would favor tumor cell growth [134,135], the decreased XOR activity observed in highly aggressive cancer cells appears to exert unexpected effects on cancer cell differentiation that also favors tumorigenesis and metastasis.

High levels of XOR were found to repress and low levels of XOR to stimulate BC cell aggressiveness *in vitro* as measured by increases in COX-2, MMP-1 secretion, and *in vitro* migration rate [136], and this appears to reflect the unexpected role played by XOR in BC cell differentiation. XOR activity was found to modulate expression of the inhibitor of differentiation protein, Id1. High levels of Id1 have been linked to highly aggressive and metaplastic BC, and the inhibition of Id1 by epithelial XOR was postulated to reduce BC aggressiveness and/or metastasis. Furthermore, loss of XOR expression in BC cells *in vitro* resulted in stimulation of Id1 level and increased BC cell aggressiveness [137]. Remarkably, epithelial XOR has now been found to modulate three of the critical signature genes mediating BC aggressiveness and metastasis: COX-2, MMP-1, and Id1 [136,137] consistent with the observed increase in clinical recurrence and metastasis in poor XOR expressing cancers. These data indicate that systemic pharmacological inhibition of XOR with the goal of reducing SUA might exacerbate tumorigenesis or metastasis by inhibition of tumor cell XOR.

**Figure 2:**
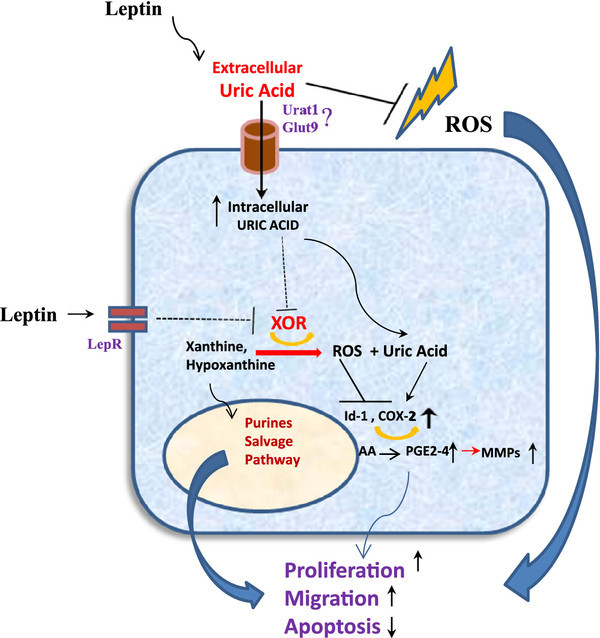
**Elevated UA and reduced intracellular XOR contribute to tumor cell proliferation, migration, and survival.** ROS scavenging properties of extracellular UA are postulated to promote cancer cell growth and survival in part by protecting cells from oxidative stress induced apoptosis. This arises because tumor cells in general exhibit poor capacity to survive oxidative stress compared with normal cells and may therefore be protected by the antioxidant ROS scavenging properties of UA (J-Shaped dose–response curve; [32]). Loss of XOR expression in the most aggressive cancer cells also contributes to tumor cell proliferation, migration, and survival. In cells showing high level XOR expression, XOR modulates COX-2 and MMP expression reducing migratory activity [136]. However, loss of XOR expression in cancer cells increases COX-2 levels, MMP expression, and migratory activity. Loss of XOR expression may arise for many reasons, including the entry of UA into cancer cells. Import of UA into XOR deficient cancer cells may further promote proliferation and survival in part by stimulating expression of COX-2. The diminished XOR expression found in aggressive cancer cells would result in shunting the XOR substrates hypoxanthine and xanthine into the salvage pathway, providing substrates for nucleotide synthesis, tumor growth, and proliferation. The independent effects of leptin on cancer cells notwithstanding [85,113], the elevated levels of leptin observed in MetS associated cancer may also drive these processes both by inducing hyperuricemia and by down regulating cancer cell XOR.

Little is known about the response of cancer cells themselves to UA. UA present in the tumor microenvironment may contribute to tumorigenesis or metastasis in ways that are distinct from the pro-inflammatory processes elicited by SUA. Exposure of human mammary cancer cells or mouse mammary epithelial cells *in vitro* to a wide concentration range of UA dose dependently increased migratory rate of both cells [136]. Migratory rate of both cells was significantly increased at even low normal levels of UA. Thus, treatment of BC cells with exogenous UA *in vitro* appeared to replicate the state of cells from more aggressive tumors with increased metastasis that have been associated with aggressive breast and colorectal cancer in patients with MetS [23,138,139]. It was postulated that the high levels of SUA observed in obesity, T2DM, and MetS may repress tumor cell XOR and inhibit its function in promoting epithelial cancer cell differentiation [136], and it has been shown that physiological levels of UA can indeed repress XOR activity [140]. However, it can be imagined as well that highly aggressive tumor cells that are naturally deficient in XOR expression [134-136] may be both poorly set up to promote differentiation but still capable of responding to exogenous UA by increased aggressiveness. Furthermore, we postulate that the elevated levels of leptin found in obesity, T2DM, and MetS may collaborate with UA and contribute to tumorigenesis and metastasis by down regulation of tumor cell XOR. Taken together these data suggest a mechanism by which diminished tumor cell XOR in conjunction with hyperuricemia and elevated leptin promote cancer cell proliferation, migration, and survival (Figure 2).

### The special cases of leukemia and tumor lysis syndrome

Certain leukemias and the Tumor Lysis Syndrome are cancer states that are also associated with severe hyperuricemia. In these cases, the hyperuricemia arises as a result of cell lysis, release of purines, and subsequent UA accumulation. XOR plays a prominent role in both cancer types as the source of UA, and current therapies are directed at inhibition of XOR with allopurinol or Febuxostat and degradation of the SUA using recombinant uricase (Rasburicase). Recent reviews provide excellent discussion of both cancer states and therapeutic strategies for reducing the associated hyperuricemia [141,142].

### UA as a signaling molecule

Low physiological levels of UA stimulated mammary cancer cell aggressiveness *in vitro*, and in cells expressing UA transport proteins capable of importing UA, UA derived from the serum, tumor associated adipocytes, macrophages, or other cells may also stimulate tumorigenesis and/or metastasis. At the present time, very little is known about the mechanisms by which UA may signal to cancer cells. However, some key observations have been generated from non-cancerous cells that may provide a useful background for further studies conducted on cancer cells themselves.

#### Renal epithelial cells

Uptake of UA by primary renal proximal tubule epithelial cells (PTEC) of the rabbit inhibited *in vitro* cell proliferation that was mediated by at least two signaling mechanisms [143]. Cell proliferation was inhibited over a broad concentration range of UA, achieved significance at 100 μM, and was profound at the hyperuricemic level of 500 μM. Co-treatment of PTEC with pharmacological inhibitors demonstrated that the inhibition of PTEC proliferation by UA was mediated by the transient activation of MAP Kinases p38, JNK, and ERK1/2. This effect was apparently transduced by UA activation of protein kinase C, cytoplasmic phospholipase A2, and NF-κB that was interpreted to comprise divergent pathways. A more complete picture of the response of PTEC to UA was obtained using a proteomic approach for UA treated HK-2 cell cultures [69]. Stable heavy isotope labeling of HK-2 cells was achieved using [13 C6]-Lys (heavy) labeling combined with exposure to 500 μM UA. Cells were mixed with control cells (no UA and Lys light), proteins prepared, trypsin digested, fractionated by liquid chromatography-mass spectrometry (LC-MS), and quantified by the relative ratio of isotopic peptide pairs. After correction for false discovery rate 782 proteins (of 13,652 peptides) were found altered by exposure to UA. Pathway network and functional group analysis identified 42 proteins associated with cell proliferation and 49 with apoptosis placing MAPK and NF-κB signaling networks at the center of UA signaling in PTEC. While details of this analysis remain to be fully deciphered, they establish the primacy of proliferation and apoptosis pathways regulated by UA in renal PTEC.

#### Vascular Smooth Muscle Cells

Perhaps the earliest observation that UA signaling may be involved in cell proliferation was generated by the observation that primary rat aortic vascular smooth muscle cell (VSMC) proliferation was stimulated by UA over a concentration range of 50 to 300 μM, an effect transduced in part by UA activation of cMyc and PDGF A-chain [144]. Unlike the human VSMC, rat VSMC do not express the URAT1 transporter or members of the OAT family, and a voltage sensitive transporter (possibly GLUT9 or URAT) may instead mediate UA induced proliferation [145]. Careful preparation of crystal free UA enabled the identification of signaling pathways induced by UA in rat VSMC [61]. NF-κB, AP-1, and the MAP kinases p38 and ERK-1/2 were activated by UA at levels from 100 to 700 μM. In addition to its effects on VSMC proliferation, UA was also found to activate the pro-inflammatory mediators MCP-1 and COX-2 [145].

#### Adipocytes

In differentiated mouse 3 T3-L1 derived adipocytes, but not undifferentiated 3 T3-L1 cells, UA was found to induce phospho-activation of p38 and ERK-1/2 MAP Kinases by a pathway involving activation of the NADPH oxidase and ROS generation [63]. Induction proceeded over a UA concentration range of 100 – 700 μM and was blocked by URAT1 inhibition with probenecid. While UA induced an apparent oxidative and nitrosative stress, it was also found subsequently to activate the inflammatory state of these cells [67]. UA at both normo- and hyperuricemic levels simultaneously increased steady state mRNA expression of the leukocyte chemokine MCP-1 and decreased expression of the anti-inflammatory protein adiponectin, and these data were largely replicated in an *in vivo* mouse model. Induction of MCP-1 was sensitive to both superoxide scavenging and apocynin inhibition as well as the PPARγ agonist rosiglitazone suggesting the involvement of the NADPH oxidase/ROS system and PPARγ, while inhibition of adiponectin appeared to involve PPARγ alone. These observations are consistent with the observed role of intracellular XOR in promoting 3 T3-L1 differentiation and adipogenesis *in vivo* in mice, an effect that was mediated in part by regulation of PPARγ activity [146]. While XOR dependent redox mechanisms were implicated in adipogenesis, the potential impact of UA *per se* on adipocyte differentiation has not been determined.

#### Leukocytes

While leukocytes exhibit a well-characterized inflammatory response to MSU crystals [10,59], exposure of RAW264.7 mouse macrophages and differentiated human U937 cells to pH adjusted and crystal free UA revealed a dramatic effect on macrophage inflammatory polarization [147]. UA from 30 to 1000 μM dose dependently modulated levels of the macrophage M2 polarization markers Arginase-1, CD36, and CD206, and this effect was transduced in part by inhibition of PPARγ sumoylation, an effect that promoted the inflammatory M1 state. Although the identity of the UA transporter mediating the response to UA was not identified, GLUT9 is expressed on leukocytes and remains a reasonable candidate for this function [148]. As an endogenous product of leukocytes, XOR activity exerts many effects on inflammatory potential, cytokine synthesis, and lipid uptake [147,149,150]. Because treatment of mouse macrophages with oxonic acid, an inhibitor of uricase, replicated many of the effects of exogenous UA, it was postulated that intracellular UA was in part responsible for the effects of endogenous XOR generated UA on leukocyte function.

#### UA as an Intracellular Redox Signal

While the detailed mechanism by which UA contributes to intracellular signaling networks is unknown, it has been postulated to involve intracellular redox dependent mechanisms. For example, in 3 T3-L1 derived adipocytes UA uptake was associated with intracellular ROS accumulation, and inhibition with apocynin implicated the NADPH oxidase as a source of ROS and placed ROS generation upstream of NF-κB, p38, and ERK-1/2 MAP Kinase activation [63,67]. These observations dovetail well with data generated in renal epithelial cells that identified several redox sensitive components of the upstream network mediating MAPK activation that are induced by UA [69]. For example, RAC1, MAPK1, MAP2K, MAP4K were all induced by UA and are known to exhibit redox-sensitive activation that in turn promotes phospho-activation of p38 and ERK-1/2 MAPK [151]. Although not well understood, induction of intracellular UA accumulation in pancreatic cancer cells (PANC-1) following radiation exposure or treatment with 5-fluorocracil was found to mediate the induction of MHC class-I related proteins MICA/B that in turn promoted sensitivity to NK-92 cell killing [152]. While the signaling mechanism responsible for the inductive effect of UA on MICA/B expression were not identified, marked elevation in MICA/B protein levels were clearly evident that were inhibited by treatment with allopurinol *in vitro*.

## Conclusions

**Figure 3:**
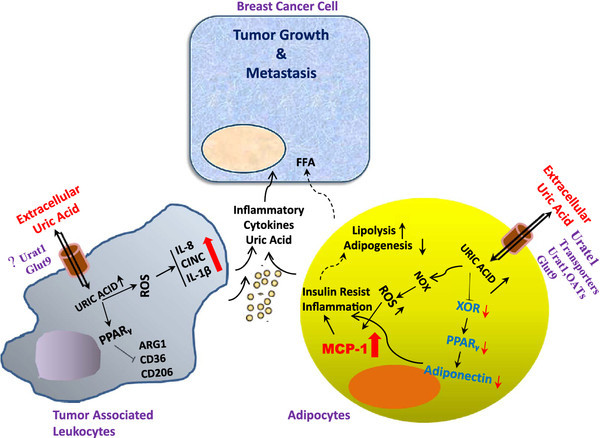
**Hypothesis for the protumorigenic role of UA in the breast cancer microenvironment.** UA is postulated to enter tumor associated and/or distant adipocytes through a UA-specific transporter (likely URAT1) where it activates the NADPH Oxidase (NOX), generating ROS. As observed in other inflammatory environments, UA at both normo- and hyperuricemic levels may simultaneously increase steady state mRNA expression of the leukocyte chemokine MCP-1 and decrease expression of the anti-inflammatory protein adiponectin. In addition, UA entering adipocytes may down-regulate expression of XOR which is known as a crucial upstream regulator of PPAR-γ, a master regulator of adipogenesis and adiponectin expression. Furthermore, as previously shown, UA may reduce levels of the macrophage antiinflammatory markers Arginase-1, CD36, and CD206, an effect transduced in part by inhibition of PPARγ sumoylation that in turn promotes macrophage inflammatory activation. Presently, the identity of the UA transporter mediating macrophage response to UA has not been identified. As an endogenous product of leukocytes, XOR activity may exert many effects on inflammatory potential, cytokine synthesis, and lipid uptake. Together, these findings support the hypothesis that hyperuricemia might be partially responsible for the low grade inflammation present in the breast tumor microenvironment that contributes to tumor cell proliferation and metastasis.

Two risk factors for the development and progression of breast cancers that are amenable to life style modification are chronic inflammation and the metabolic syndrome. To ameliorate inflammation, clinical trials abound that focus on COX-2 inhibition. The principal factors felt to mediate cancer risk from metabolic syndrome have included insulin resistance, high IGF1R signaling and higher tissue levels of estrogen (for breast cancer). This review proposes two new targets that may mechanistically integrate inflammation and metabolic syndrome, have been largely ignored, and are known to be druggable. Realization of the important role played by UA as a signaling molecule mediating the inflammatory effects of hyperuricemia in adipocytes and leukocytes, as well as in signaling to cancer cells (Figure 3) has emphasized the relevance of managing UA therapeutically which could significantly improve treatment strategies for cancer that is associated with hyperuricemic disorders.

Significant side effects of systemic chronic inhibition of XOR have been recognized for many years. Confounding problems associated with chronic inhibition of XOR include diarrhea, diminished renal function, leukopenia, hypersensitivity reactions, vasculitis [3], and even exacerbation of vascular injury through recently identified effects on nitrite reduction [153]. While the frequency of allopurinol hypersensitivity syndrome may be significantly reduced by avoiding the administration of allopurinol to subjects bearing the HLA-B58 haplotype [154], it remains a serious syndrome with high mortality. Systemic pharmacological inhibition of cancer cell XOR could theoretically exacerbate tumorigenesis, metastasis, and mortality. These unwanted side effects underscore the urgent need for mechanism based pre-clinical studies that can identify optimal strategies for management of hyperuricemia in relevant cancer models. Recently developed conditional knockout models for XOR that are based on CRE/Lox technology may be of particular value for the knockdown of XOR in specific target cells, such as hepatocytes or adipocytes, that are principal sources of SUA [137]. Likewise, similar CRE/Lox technology applied to the GLUT9 transporter [155] will be highly valuable in delineating the role of SUA in pre-clinical models of human cancer as well. The goal of specifically managing SUA without exacerbating tumorigenesis and/or metastasis will undoubtedly involve novel therapeutic strategies, but these efforts could significantly improve therapeutic strategy for cancer associated with obesity, T2DM, and MetS.

## Abbreviations

UA: Uric Acid; SUA: Serum Uric Acid; XOR: Xanthine Oxidoreductase; T2DM: Type 2 Diabetes Mellitus; MetS: Metabolic Syndrome; MAPK: Mitogen Activated Protein Kinase; ROS: Reactive Oxygen Species; VSMC: Vascular Smooth Muscle Cell; CVD: CardioVascular Disease; BC: Breast Cancer; CRC: Colorectal Cancer; CRP: C-Reactive Protein; MSU: Monosodium Urate.

## Competing interests

The authors declare no financial or non-financial competing interests. Dr. Johnson holds patent applications related to lowering uric acid in the treatment of metabolic syndrome, kidney disease and hypertension, and has consulted for Ardea, Novartis, Danone, and Astellas. He also holds a patent for the use of allopurinol to treat primary hypertension with the University of Washington and Merck, Inc. Dr. Wright holds a USA clinical use patent (10/573,354) for modulating XOR in diverse inflammatory settings.

## Authors’ contributions

The authors contributed equally to the research, writing, and review of this manuscript. All authors read and approved the final manuscript.
